# Timing of Remdesivir Initiation and Clinical Outcomes in Hospitalized Patients with COVID-19 Who Are at High Risk of Disease Progression in Japan: A Health Insurance Claims Database Study

**DOI:** 10.3390/v18040479

**Published:** 2026-04-21

**Authors:** Yuichiro Shindo, Yi Piao, Mark Berry, Heribert Ramroth, Manami Yoshida

**Affiliations:** 1 Department of Respiratory Medicine, Nagoya University Graduate School of Medicine, Nagoya 466-8550, Japan; shindo.yuichiro.y7@f.mail.nagoya-u.ac.jp; 2 Gilead Sciences, K.K., 16/F GRAN TOKYO SOUTH TOWER, 1-9-2 Marunouchi, Chiyoda-ku, Tokyo 100-6616, Japan; 3 Gilead Sciences, Inc., 333 Lakeside Dr., Foster City, CA 94405, USA; 4 Gilead Sciences, EU, 2 Roundwood Ave., Uxbridge UB11 1AF, UK

**Keywords:** COVID-19, remdesivir, hospitalized patients, high-risk patients, initiation timing, DeSC database

## Abstract

Early initiation of remdesivir (RDV) is recommended to improve COVID-19 outcomes, but real-world studies describing patterns of RDV use and related outcomes among Japanese COVID-19 patients at high-risk of severe outcomes or death are limited. This claims-based cohort study included 60,165 high-risk patients hospitalized with COVID-19 between October 2021 and June 2023 using the DeSC Healthcare claims database. Patients were categorized into early-RDV (within 2 days of hospital admission), late-RDV (between day 3 and day 7), and no-RDV groups based on RDV initiation timing. Descriptive analyses were performed according to RDV groups. Of the study patients, ≥85% were very elderly (≥75 years). Approximately 39% of patients received early RDV, 2% received late RDV, and 59% received no RDV. By day 28, the proportion of alive discharge for early-, late-, and no-RDV groups was 74.9%, 63.1%, and 71.8%, respectively. The mortality for early-, late-, and no-RDV groups was 7.7%, 8.8%, and 8.4%, respectively. Future hypothesis-driven studies with an appropriate adjustment for confounders are needed to formally evaluate the impact of RDV initiation timing on clinical outcomes in this high-risk, predominantly late-elderly population in Japan.

## 1. Introduction

Since the start of the COVID-19 pandemic, there have been approximately 760 million cumulative cases and 6.9 million deaths reported worldwide as of May 2023 [1]. By May 2023, Japan had reported nearly 34 million COVID-19 infections and nearly 75,000 deaths [2], with 30,000 deaths continuing to occur annually. Although the death rate in Japan is significantly lower compared to the global average, the absolute number of deaths remains alarming and underscores the ongoing need for effective interventions. Importantly, individuals over the age of 70 accounted for more than 90% of severe COVID-19 cases and more than 70% of mortalities [3].

Remdesivir (RDV) was first approved in Japan on 7 May 2020, which was one week after the approval by the US Emergency Use Authorization [4]. It is recommended as an antiviral therapy for treatment of hospitalized patients with COVID-19 by various guidelines, including those from the US National Institutes of Health (NIH) [5], the Infectious Diseases Society of America (IDSA) [6], the World Health Organization (WHO) [7], and multiple Japanese medical societies, including the Japanese Association for Infectious Diseases and the Japanese Respiratory Society [8]. Notably, the most recent 2025 Clinical Management Guidelines for COVID-19 in Japan emphasize the importance of early diagnosis and early treatment in managing viral infectious diseases and reducing the burden of hospitalization [8]. Early antiviral treatment is considered particularly important for high-risk patients, defined as those who have a higher risk of developing severe COVID-19 outcomes or death, such as older adults and those with immunocompromised conditions [5]. The early timing of RDV administration has been supported by both clinical trials and observational studies [9,10,11,12,13,14]. Most of these studies were conducted in younger or mixed-age populations, in which very elderly patients were either underrepresented or analyzed only as subgroups. However, the demographic context of COVID-19 hospitalizations in Japan is distinct from that of many other countries. Hospitalized patients with COVID-19 in Japan are predominantly late-elderly (aged ≥ 75 years), among whom advanced age itself represents a high-risk factor [3].

Despite the availability of guideline recommendations, how RDV is actually used in routine clinical practice—including the frequency of non-use and the timing of initiation—remains poorly described in an aging society in Japan. Furthermore, the outcomes most relevant to late-elderly patients, such as discharge status, length of hospital stay, and healthcare utilization, are underexplored. Real-world descriptive data on RDV utilization patterns in this population are, therefore, of particular value. Although a large registry-based observational study in Japan suggested that RDV initiated within a few days of hospital admission was associated with better clinical outcomes compared with no RDV treatment [15], little is known about how early, late, or no RDV initiation is distributed among high-risk, predominantly late-elderly hospitalized patients in Japan.

This study aims to address this gap by using a large claims database in Japan to investigate the real-world pattern of RDV initiation timing (early, late, and no) and describe clinical outcomes and healthcare utilization among COVID-19 patients at high risk of disease progression. Given that key clinical variables in this population—including frailty, functional status, and disease severity indicators—are often not captured in administrative claims data, risk-adjusted causal analyses are particularly challenging. This study was, therefore, intentionally designed as a descriptive analysis to provide insights into current treatment practices and to inform future hypothesis-driven and risk-adjusted research and clinical strategies.

## 2. Materials and Methods

### 2.1. Study Design and Data Source

This was a claims database study using the DeSC Healthcare database, a commercially available, patient-level administrative claims and health checkup database in Japan that covers approximately 10% of the general population, provided by DeSC Healthcare, Inc. [16]. It anonymously collects a wide range of information, including individual unique identifiers, demographic variables, diagnoses coded according to the standard disease name codes, medical procedures, prescriptions, and medical costs [16].

Among these patients, approximately 19% were covered by the National Health Insurance for self-employed, unemployed, and retired individuals; approximately 2% were covered by the Health Insurance Societies for employees of large companies and their dependents; and approximately 79% were covered by the medical care system for the late-elderly (aged 75 years and above) in Japan.

### 2.2. Patients

We included patients hospitalized with a COVID-19 infection between 1 October 2021 and 9 June 2023 (see Table S1 for the definitions of COVID-19 using ICD-10 codes). The index date was defined as the day of hospital admission. Patients were included if they had a diagnosis of COVID-19 (primary or secondary) recorded in the same calendar month; patients whose initial COVID-19 diagnosis occurred after the admission were excluded. Patients were further included if they met the following criteria: (1) were aged ≥ 75 years, or ≥18 years with at least one risk factor for severe disease as defined by the Centers for Disease Control and Prevention (CDC) [17]; (2) had continuous insurance enrollment for at least 84 days before and including the index date, with no single gap in enrollment exceeding 28 days; (3) had the start date of any COVID-19-related medical care on the same date as or prior to hospital admission; (4) had no RDV use and no evidence of pregnancy (pregnancy was defined as the 10 months preceding the child’s date of birth, based on the recorded birth date) within 84 days prior to the index date; (5) had no missing information on age, sex, or geographic region.

Definitions for patient characteristics, including oxygen supply levels, COVID-19–related therapeutic agents, and comorbidities, are detailed in Tables S2–S4.

### 2.3. Exposure

COVID-19 is characterized by viral replication early in its course [18]; therefore, prompt antiviral therapy is recommended by various guidelines [6,8]. While the RDV label does not specify timing, the NIH guidelines suggest greater benefits are associated with administration within 7–10 days of symptom onset [5]. Moreover, real-world evidence indicates improved outcomes within 2 days of admission [12]. Accordingly, the timing of RDV initiation was categorized as follows: (1) early RDV, initiation on or before day 2 of hospital admission; (2) late RDV, initiation between day 3 and day 7 (inclusive); (3) no RDV, no RDV administration during hospital stay. Patients whose RDV treatment was initiated on or after day 8 were considered to have received delayed RDV administration, likely due to special circumstances such as injuries. Accordingly, these patients were not included in the analyses.

### 2.4. Outcomes

The exploratory outcomes of interest were as follows: clinical outcomes by RDV groups, including vital status at discharge by day 28, length of hospital stay (LOS), medical costs, and hospital readmission within 28 days and 56 days after the index date.

Alive discharge was defined as discharge from hospitals within 28 days of the index date. Death within 28 days was identified using claims data and reasons of disqualification in insurance register data. Since this dataset provides only the month and year of death (not specific dates), the date of death was estimated as the last claim date within the month of the death.

### 2.5. Statistical Analysis

Descriptive statistics were used in this study. Patient characteristics were summarized by RDV groups, with age (a continuous variable) reported as the mean and standard deviation, and categorical variables reported as proportions. Each event of vital status at discharge was estimated using data from Municipalities-based National Health Insurance and the Medical Care for Elderly People, where mortality information was available. Alive discharge and death by day 28 were summarized by RDV groups. We calculated the percentages and the corresponding 95% confidence intervals (CIs).

We also summarized the LOS, medical costs, and readmissions by RDV groups. The LOS was defined as the number of days from hospital admission to discharge. We calculated the average LOS by capping LOS values at 28 days and then taking the mean across all patients with a discharge date to avoid interpretation issues caused by extreme outliers. Cumulative medical costs through day 84 were estimated for patients with continuous insurance coverage by summing all outpatient, inpatient, and pharmacy claims, and the results are presented as mean costs. Readmissions within 28 days and 56 days after the index date were calculated among patients with insurance coverage through day 28 and day 56, respectively.

All analyses were performed using Microsoft Excel and SAS version 9.4 (SAS Institute Inc., Cary, NC, USA).

## 3. Results

### 3.1. Timing of RDV Initiation and Patient Characteristics

**Figure 1:**
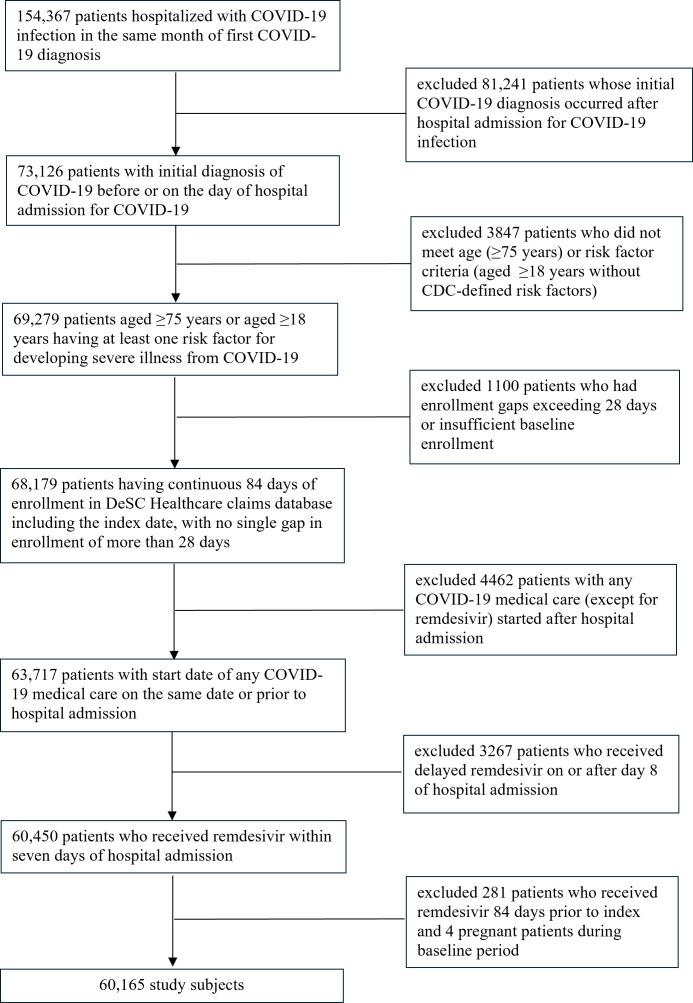
Flow chart of patient selection. Data were collected during the period from January 2020 to August 2023. The baseline period was defined as three months before the index date.

**Figure 2:**
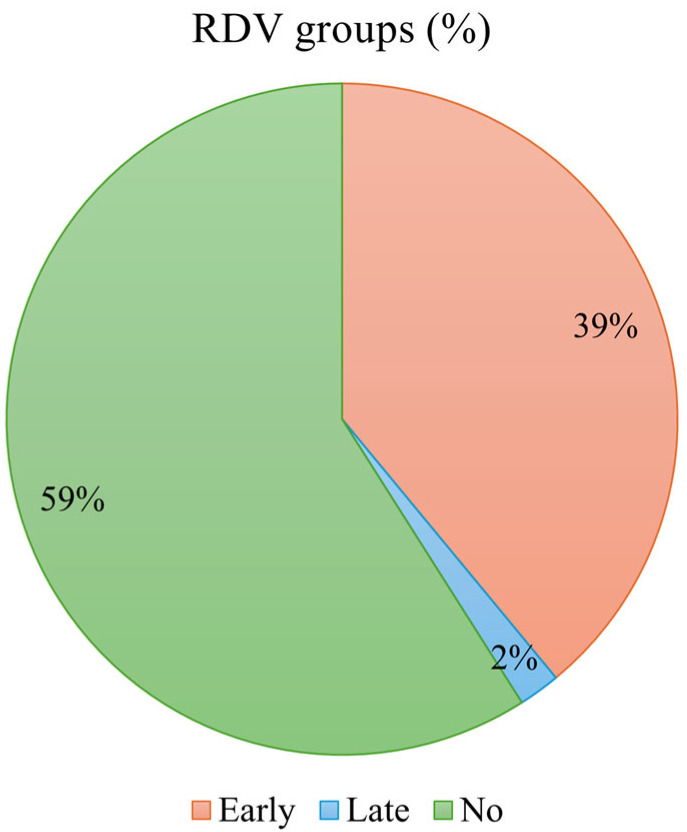
Percentage of patients by RDV groups.

The study sample included 60,165 hospitalized COVID-19 patients (Figure 1). Among these, approximately 39% of patients were categorized as the early-RDV group, 2% as the late-RDV group, and 59% received no RDV (Figure 2).

The study population was predominantly very elderly; the mean age was 84.0, 82.8, and 82.8 years in the early-, late-, and no-RDV groups, respectively, with over 85% of patients aged ≥ 75 years across all groups. The proportion of female patients was 49.6%, 43.3%, and 51.9% in the early-, late-, and no-RDV groups, respectively. Over 96% of patients across all groups were hospitalized during the Omicron period (Table 1).

The median time from initial diagnosis to hospital admission was 0 days across all groups. The proportion of patients admitted to acute care hospitals was 16.3% in the early-RDV group, 20.5% in the late-RDV group, and 15.4% in the no-RDV group (Table 1). The proportion of patients with hypertension was approximately 69%, 70%, and 72% in the early-, late-, and no-RDV groups, respectively.

### 3.2. Vital Status at Discharge by Timing of RDV Initiation

By day 28, the proportion of alive discharge was 74.9% in the early-RDV group, 63.1% in the late-RDV group, and 71.8% in the no-RDV group (Table 2). The corresponding day 28 mortality was 7.7% in the early-RDV group, 8.8% in the late-RDV group, and 8.4% in the no-RDV group (Table 2).

### 3.3. LOS, Medical Cost, and Readmission by Timing of RDV Initiation

The mean LOS was 12.6 days in the early-RDV group, 16.0 days in the late-RDV group, and 13.2 days in the no-RDV group (Table 3). The mean cumulative medical cost through day 84 was 2,206,795 yen in the early-RDV group, 2,719,162 yen in the late-RDV group, and 1,692,828 yen in the no-RDV group (Table 3). By day 56, the proportion of readmission was 10.7% among patients in the early-RDV group, 13.4% in the late-RDV group, and 10.5% in the no-RDV group (Table 3).

## 4. Discussion

This study provides real-world evidence on different patterns of RDV initiation timing and the corresponding unadjusted clinical outcomes among patients hospitalized with COVID-19 in Japan, with a particular focus on those who are at high risk for disease progression, such as elderly patients and patients with comorbidities.

Among 60,165 high-risk hospitalized patients, approximately 39% received early RDV (within 2 days of admission), 2% received late RDV (days 3–7), and 59% received no RDV during their hospital stay. This distribution indicates that a substantial proportion of high-risk, late-elderly hospitalized patients did not receive RDV, which represents a gap from the recommendations of treatment guidelines from Japanese medical societies, which recommend RDV treatment for eligible hospitalized patients [8]. These findings highlight the need for ongoing interventions to improve adherence to the guidelines. Future studies are needed to explore factors contributing to this gap between guideline recommendations and real-world clinical practices.

This study was intentionally designed as a descriptive analysis. Key clinical variables that are known to influence both treatment decisions and patient outcomes—including symptom onset date, vaccination status, peripheral oxygen saturation (SpO_2_), and functional or frailty measures—were not available in the DeSC claims database. Conducting risk-adjusted or causal analyses without these variables can lead to misleading estimates of treatment effect. Accordingly, all outcome observations reported in this study should be interpreted as descriptive patterns reflecting the characteristics of patients who received different treatment approaches in routine clinical practice, and not as evidence of the effectiveness or causal impact of RDV initiation timing on clinical outcomes.

The principal value of this study lies in its foundational, descriptive assessment of real-world treatment practices in a healthcare system dominated by late-elderly patients—a population that has been underrepresented in prior RDV research. The characterization of clinical outcomes as well as healthcare utilization patterns—including discharge status, length of hospital stay, medical costs, and readmission rates—across different treatment groups may help improve clinical education and inform future research design in an aging society. In particular, future research should identify the clinical and patient-level factors driving RDV initiation decisions and define outcomes—such as discharge status and care transitions—that are clinically meaningful for very elderly populations, as well as conduct effectiveness studies using data sources that capture frailty, disease severity, and symptom onset timing to formally evaluate the impact of RDV initiation timing on outcomes through appropriately adjusted causal analyses.

Several limitations should be noted. First, as described above, this study is descriptive in nature and baseline differences between RDV groups were not adjusted for; therefore, differences in outcomes cannot be interpreted causally. Second, patient characteristics were described with several variables, but variables that may affect outcomes, such as date of symptom onset, vaccination status, and indicators of disease severity (e.g., SpO_2_), were not included due to unavailable data. Third, because the database only provides the month and year of death, the exact date of death was estimated from the last claim date within the month of death, which may have introduced minor inaccuracies in the timing of mortality outcomes. However, as this limitation applies equally across all RDV groups, it is unlikely to have materially affected the descriptive patterns observed. Fourth, this database may not be representative of the Japanese population, as some characteristics—most notably the very high proportion of very elderly patients—do not fully reflect the national data. Furthermore, the very small percentage of individuals below 75 years of age likely underestimates the actual proportion in the overall population [19]. Finally, our study may be subject to survival bias, as only patients who survived long enough to potentially receive RDV treatment were included in the late-RDV group. As a result, this may have influenced the observed outcomes. Given these constraints, the study’s descriptive findings may not be generalizable to all high-risk hospitalized COVID-19 patients and should not be interpreted as evidence of a causal effect of RDV initiation timing on clinical outcomes.

## 5. Conclusions

In this large claims-based study of 60,165 high-risk hospitalized COVID-19 patients—predominantly late-elderly—nearly 40% of patients received RDV early, while more than half of the patients did not receive RDV at all. These findings reveal a substantial gap between guideline recommendations and real-world antiviral practice in this population. Observed alive discharge rates by day 28 were 74.9%, 63.1%, and 71.8% in the early-, late-, and no-RDV groups, respectively; corresponding mortality rates were 7.7%, 8.8%, and 8.4%. As this study was intentionally descriptive and key clinical variables were unavailable, observed outcome patterns across RDV groups should not be interpreted as evidence of treatment effects. Future research with appropriate covariate data is needed to clarify RDV timing effects on outcomes in late-elderly patients with COVID-19.

## Figures and Tables

**Table 1:** Baseline characteristics of hospitalized patients with COVID-19 who are at high risk of disease progression.

Characteristic, %	Early (n = 23,234)	Late (n = 1251)	No (n = 35,680)
Mean age, years (SD)	84.0 (8.7)	82.8 (9.2)	82.8 (10.2)
Aged 75+	91.0	86.9	87.0
Female sex	49.6	43.3	51.9
Number of beds ≥ 500	20.9	25.4	19.0
University hospital	5.7	8.3	4.9
Acute care hospital	16.3	20.5	15.4
Omicron (Jan 2022–Jun 2023)	99.9	99.2	96.9
Time from initial diagnosis to index, Days (SD)	0.8 (1.8)	0.6 (1.6)	1.1 (3.1)
Other COVID-19 treatment prior to index	3.0	2.1	2.9
Oxygen level at index			
No supplemental oxygen charges	58.6	67.0	75.7
LFO	38.5	31.1	21.5
HFO	1.7	0.8	0.7
IMV/ECMO	1.2	1.1	2.1
Solid malignancy	15.6	19.9	18.6
Type II diabetes	37.5	39.5	39.1
Cerebrovascular disease	30.8	31.8	29.6
Heart failure	36.1	37.3	39.3
Ischemic heart disease	22.0	26.1	25.5
Hypertension	68.6	69.9	71.8
Bronchial asthma	13.8	14.1	12.9
Chronic obstructive pulmonary disease	5.6	4.1	4.8
Chronic kidney disease with dialysis	7.9	11.8	12.3
Mood disorder	10.7	11.0	9.8
Schizophrenia	10.1	10.6	8.3
Dementia	24.4	22.0	19.4

Abbreviations: ECMO, extracorporeal membrane oxygenation; HFO, high-flow oxygen; IMV, invasive mechanical ventilation; LFO, low-flow oxygen; SD, standard deviation. Data are presented as % unless otherwise indicated.

**Table 2:** Vital status at discharge by timing of RDV initiation in hospitalized patients with COVID-19 who are at high risk of disease progression.

Outcomes	Early	Late	No
Number of patients	21,998	1181	33,380
Alive discharge			
Number of events	16,471	745	23,979
% discharged (95% CI)	74.9 (74.3, 75.4)	63.1 (60.3, 65.8)	71.8 (71.4, 72.3)
Mean time to alive discharge (days)	18.9	26.6	20.4
Death			
Number of events	1698	104	2819
% death (95% CI)	7.7 (7.4, 8.1)	8.8 (7.2, 10.4)	8.4 (8.1, 8.7)
Mean time to death (days)	26.8	26.8	26.5

Abbreviations: CI, confidence interval. Data are presented as numbers unless otherwise indicated.

**Table 3:** Length of stay, medical costs, and readmission by timing of RDV initiation in hospitalized patients with COVID-19 who are at high risk of disease progression.

Outcomes	Early	Late	No
LOS			
Number of patients	22,473	1212	35,680
Mean (days)	12.6	16.0	13.2
Medical cost			
Number of patients	18,006	970	28,310
Total cost (1000 Yen)	39,735,542	2,637,587	47,923,950
Mean cost (1000 Yen)	2207	2719	1693
Readmission in 28 days			
Number of patients	21,468	1159	33,745
Number of events	1078	56	1345
% readmission	5.0	4.8	4.0
Readmission in 56 days			
Number of patients	19,861	1052	31,319
Number of events	2126	141	3295
% readmission	10.7	13.4	10.5

Number of patients differed by each outcome according to the prespecified inclusion criteria. Abbreviations: LOS, length of stay.

## Data Availability

The data that support the findings of this study are available from DeSC Healthcare, Inc., but restrictions apply to the availability of these data, which were used under license for the current study, and so are not publicly available. Data are, however, available from the authors upon reasonable request and with permission of DeSC Healthcare, Inc.

## Supplementary Materials

The following supporting information can be downloaded at: https://www.mdpi.com/article/10.3390/v18040479/s1, Table S1: Definition of COVID-19 using ICD-10 codes; Table S2. Classification of oxygen support level; Table S3. List of COVID-19-related therapeutic agents; Table S4. Medical conditions and associated codes identified by the Centers for Disease Control and Prevention (CDC) as risk factors for severe illness or death from COVID-19.

## Abbreviations

The following abbreviations are used in this manuscript:

CI	Confidence intervals
ECMO	Extracorporeal membrane oxygenation
HFO	High-flow oxygen
IMV	Invasive mechanical ventilation
LFO	Low-flow oxygen
LOS	Length of hospital stay
NIH	National Institutes of Health
RDV	Remdesivir
