# Supplementary material for: Timing of Remdesivir Initiation and Clinical Outcomes in Hospitalized Patients with COVID-19 Who Are at High Risk of Disease Progression in Japan: A Health Insurance Claims Database Study
Source: Viruses. 2026 Apr 21;18(4):479. doi: 10.3390/v18040479 (PMC13120663; doi:10.3390/v18040479)
Supplement: Supplementary file 1 [file viruses-18-00479-s001.zip › viruses-4178374-supplementary.pdf]

Supplementary Material

Table S1. Definition of COVID-19 using ICD-10 codes

ICD-10	COVID-19-related infection
B34.2	Coronavirus infection, unspecified site
	MERS
U07.1	COVID-19, virus identified
U07.2	COVID-19, virus not identified
U08	Personal history of COVID-19
U08.9	Personal history of COVID-19, unspecified
U09	Post COVID-19 condition
U09.9	Post COVID-19 condition, unspecified
U10	Multisystem inflammatory syndrome associated with COVID-19
U10.9	Multisystem inflammatory syndrome associated with COVID-19, unspecified

Table S2. Classification of oxygen support level

Classification	Procedure code	Claims code
LOW	J024	140005610
High	J026	140005910
High	J026-2	140037810
High	J026-3	140006050
High	J0454	140010050
High	J0454	140024250
High	J0454	140039550
High	J0454	140039650
High	J0454	140064550
High	J0454	140064750
High	J026-4	140055810
High	J026-41	140057310
High	J026-42	140057410
IMV or ECMO	J0451	140009310
IMV or ECMO	J0452	140030830
IMV or ECMO	J0452	140030930
IMV or ECMO	J0452	140031030
IMV or ECMO	J0452	140031130
IMV or ECMO	J0452	140031230
IMV or ECMO	J0452	140031330
IMV or ECMO	J0452	140031430
IMV or ECMO	J0452	140031530
IMV or ECMO	J0452	140031630
IMV or ECMO	J0452	140031730
IMV or ECMO	J0453	140023510
IMV or ECMO	J0453	140063810
IMV or ECMO	J0454	140009450
IMV or ECMO	J0454	140009550
IMV or ECMO	J0454	140009650
IMV or ECMO	J0454	140009750
IMV or ECMO	J0454	140009850
IMV or ECMO	J0454	140009950
IMV or ECMO	J0454	140010150
IMV or ECMO	J0454	140023650

IMV or ECMO	J0454	140023750
IMV or ECMO	J0454	140023850
IMV or ECMO	J0454	140023950
IMV or ECMO	J0454	140024050
IMV or ECMO	J0454	140024150
IMV or ECMO	J0454	140024350
IMV or ECMO	J0454	140039850
IMV or ECMO	J0454	140039950
IMV or ECMO	J0454	140063950
IMV or ECMO	J0454	140064050
IMV or ECMO	J0454	140064150
IMV or ECMO	J0454	140064250
IMV or ECMO	J0454	140064350
IMV or ECMO	J0454	140064650
IMV or ECMO	K6011	150147610
IMV or ECMO	K6012	150147910
IMV or ECMO	K6013	150147770
IMV or ECMO	K6013	150147870
IMV or ECMO	K6013	150347170
IMV or ECMO	K6014	150275870
IMV or ECMO	K601-2	150428410
IMV or ECMO	K601-2	150428510
IMV or ECMO	K6021	150262910
IMV or ECMO	K6022	150275710
IMV or ECMO	K916	150432610
IMV or ECMO	K916	150432710
IMV or ECMO	K916	150432810
IMV or ECMO	K916	150432970

Abbreviations: IMV, Invasive mechanical ventilation; ECMO, Extracorporeal membrane oxygenation

Table S3. List of COVID-19–related therapeutic agents

Classification	ATC code	Generic Name	Claims code
Corticosteroid	H02A1	Dexamethasone palmitate	620003832
Corticosteroid	H02A1	Dexamethasone palmitate	620525001
Corticosteroid	H02A1	Dexamethasone palmitate	620525101
Corticosteroid	H02A1	Dexamethasone palmitate	620525201
Corticosteroid	H02A1	Dexamethasone palmitate	620525301
Corticosteroid	H02A1	Dexamethasone palmitate	620525401
Corticosteroid	H02A1	Dexamethasone palmitate	620525601
Corticosteroid	H02A1	Dexamethasone palmitate	620525701
Corticosteroid	H02A1	Dexamethasone palmitate	620525801
Corticosteroid	H02A1	Dexamethasone palmitate	620525901
Corticosteroid	H02A1	Dexamethasone palmitate	640422046
Corticosteroid	H02A1	Dexamethasone palmitate	640422047
Corticosteroid	H02A1	Dexamethasone palmitate	642450007
Corticosteroid	H02A1	Dexamethasone palmitate	642450008
Corticosteroid	H02A1	Dexamethasone palmitate	642450054
Corticosteroid	H02A1	Dexamethasone palmitate	642450055
Corticosteroid	H02A1	Dexamethasone palmitate	642450056
Corticosteroid	H02A1	Triamcinolone acetonide	620004660
Corticosteroid	H02A1	Triamcinolone acetonide	620004661
Corticosteroid	H02A1	Hydrocortisone sodium succinate	620007332
Corticosteroid	H02A1	Hydrocortisone sodium succinate	620007333
Corticosteroid	H02A1	Hydrocortisone sodium succinate	620007334
Corticosteroid	H02A1	Hydrocortisone sodium succinate	620007335
Corticosteroid	H02A1	Hydrocortisone sodium succinate	620008816
Corticosteroid	H02A1	Hydrocortisone sodium succinate	620008818
Corticosteroid	H02A1	Hydrocortisone sodium succinate	620008819

Corticosteroid	H02A1	Hydrocortisone sodium succinate	620518605
Corticosteroid	H02A1	Hydrocortisone sodium succinate	620518905
Corticosteroid	H02A1	Hydrocortisone sodium succinate	620519005
Corticosteroid	H02A1	Hydrocortisone sodium succinate	640454009
Corticosteroid	H02A1	Hydrocortisone sodium succinate	642450029
Corticosteroid	H02A1	Hydrocortisone sodium phosphate	620002208
Corticosteroid	H02A1	Hydrocortisone sodium phosphate	620002209
Corticosteroid	H02A1	Hydrocortisone sodium phosphate	620005162
Corticosteroid	H02A1	Hydrocortisone sodium phosphate	620519503
Corticosteroid	H02A1	Hydrocortisone sodium phosphate	620519603
Corticosteroid	H02A1	Hydrocortisone sodium phosphate	620530402
Corticosteroid	H02A1	Hydrocortisone sodium phosphate	620530502
Corticosteroid	H02A1	Hydrocortisone sodium phosphate	642450115
Corticosteroid	H02A1	Hydrocortisone sodium phosphate	642450116
Corticosteroid	H02A1	Hydrocortisone sodium phosphate	642450169
Corticosteroid	H02A1	Hydrocortisone sodium phosphate	642450170
Corticosteroid	H02A1	Hydrocortisone sodium phosphate	642450171
Corticosteroid	H02A1	Betamethasone sodium phosphate	620002613
Corticosteroid	H02A1	Betamethasone sodium phosphate	620002614
Corticosteroid	H02A1	Betamethasone sodium phosphate	620002615
Corticosteroid	H02A1	Betamethasone sodium phosphate	620002616
Corticosteroid	H02A1	Betamethasone sodium phosphate	620002617
Corticosteroid	H02A1	Betamethasone sodium phosphate	620003829
Corticosteroid	H02A1	Betamethasone sodium phosphate	620003830
Corticosteroid	H02A1	Betamethasone sodium phosphate	620003831
Corticosteroid	H02A1	Betamethasone sodium phosphate	620006275
Corticosteroid	H02A1	Betamethasone sodium phosphate	620006276
Corticosteroid	H02A1	Methylprednisolone sodium succinate	620001310

Corticosteroid	H02A1	Methylprednisolone sodium succinate	620007356
Corticosteroid	H02A1	Methylprednisolone sodium succinate	620007357
Corticosteroid	H02A1	Methylprednisolone sodium succinate	620007358
Corticosteroid	H02A1	Methylprednisolone sodium succinate	620007359
Corticosteroid	H02A1	Methylprednisolone sodium succinate	620528103
Corticosteroid	H02A1	Methylprednisolone sodium succinate	620528502
Corticosteroid	H02A1	Methylprednisolone sodium succinate	620528505
Corticosteroid	H02A1	Methylprednisolone sodium succinate	620528901
Corticosteroid	H02A1	Methylprednisolone sodium succinate	620528905
Corticosteroid	H02A1	Methylprednisolone sodium succinate	620529204
Corticosteroid	H02A1	Methylprednisolone sodium succinate	622100401
Corticosteroid	H02A1	Methylprednisolone sodium succinate	622100501
Corticosteroid	H02A1	Methylprednisolone sodium succinate	622329500
Corticosteroid	H02A1	Methylprednisolone sodium succinate	622329600
Corticosteroid	H02A1	Methylprednisolone sodium succinate	622329700
Corticosteroid	H02A1	Methylprednisolone sodium succinate	622756500
Corticosteroid	H02A1	Methylprednisolone sodium succinate	622756600
Corticosteroid	H02A1	Methylprednisolone sodium succinate	640454024
Corticosteroid	H02A1	Methylprednisolone sodium succinate	640454025
Corticosteroid	H02A1	Methylprednisolone sodium succinate	640454026
Corticosteroid	H02A1	Methylprednisolone sodium succinate	642450049
Corticosteroid	H02A1	Methylprednisolone sodium succinate	642450050
Corticosteroid	H02A1	Methylprednisolone sodium succinate	642450139
Corticosteroid	H02A1	Methylprednisolone sodium succinate	642450140
Corticosteroid	H02A1	Methylprednisolone sodium succinate	642450141
Corticosteroid	H02A1	Methylprednisolone sodium succinate	642450142
Corticosteroid	H02A1	Methylprednisolone acetate	620007381
Corticosteroid	H02A1	Methylprednisolone acetate	620007382

Corticosteroid	H02A2	Cortisone acetate	620002513
Corticosteroid	H02A2	Dexamethasone	612450121
Corticosteroid	H02A2	Dexamethasone	620006985
Corticosteroid	H02A2	Dexamethasone	620006986
Corticosteroid	H02A2	Dexamethasone	620008651
Corticosteroid	H02A2	Dexamethasone	620521302
Corticosteroid	H02A2	Dexamethasone	621997701
Corticosteroid	H02A2	Dexamethasone	622359901
Corticosteroid	H02A2	Triamcinolone	612450070
Corticosteroid	H02A2	Hydrocortisone	620006903
Corticosteroid	H02A2	Fludrocortisone acetate	620007078
Corticosteroid	H02A2	Prednisolone	610408661
Corticosteroid	H02A2	Prednisolone	610422253
Corticosteroid	H02A2	Prednisolone	610431117
Corticosteroid	H02A2	Prednisolone	610454071
Corticosteroid	H02A2	Prednisolone	612450051
Corticosteroid	H02A2	Prednisolone	612450096
Corticosteroid	H02A2	Prednisolone	612450118
Corticosteroid	H02A2	Prednisolone	620000125
Corticosteroid	H02A2	Prednisolone	620000694
Corticosteroid	H02A2	Prednisolone	620000695
Corticosteroid	H02A2	Prednisolone	620000696
Corticosteroid	H02A2	Prednisolone	620000697
Corticosteroid	H02A2	Prednisolone	620000698
Corticosteroid	H02A2	Prednisolone	620001894
Corticosteroid	H02A2	Prednisolone	620001895
Corticosteroid	H02A2	Prednisolone	620001896
Corticosteroid	H02A2	Prednisolone	620003272
Corticosteroid	H02A2	Prednisolone	620004294
Corticosteroid	H02A2	Prednisolone	620004387
Corticosteroid	H02A2	Prednisolone	620005848
Corticosteroid	H02A2	Prednisolone	620527133
Corticosteroid	H02A2	Prednisolone	620527136
Corticosteroid	H02A2	Prednisolone	620527205
Corticosteroid	H02A2	Prednisolone	620527207
Corticosteroid	H02A2	Prednisolone	621559301
Corticosteroid	H02A2	Betamethasone	620004578
Corticosteroid	H02A2	Betamethasone	620004620
Corticosteroid	H02A2	Betamethasone	620005133
Corticosteroid	H02A2	Betamethasone	620005134
Corticosteroid	H02A2	Betamethasone	620006162
Corticosteroid	H02A2	Betamethasone	620521501
Corticosteroid	H02A2	Methylprednisolone	620005125

Corticosteroid	H02A2	Methylprednisolone	620005126
Corticosteroid	H02B0	Betamethasone and d-Chlorpheniramine Maleate	610407106
Corticosteroid	H02B0	Betamethasone and d-Chlorpheniramine Maleate	612450012
Corticosteroid	H02B0	Betamethasone and d-Chlorpheniramine Maleate	612450013
Corticosteroid	H02B0	Betamethasone and d-Chlorpheniramine Maleate	620530701
Corticosteroid	H02B0	Betamethasone and d-Chlorpheniramine Maleate	620530801
Corticosteroid	H02B0	Betamethasone and d-Chlorpheniramine Maleate	620531001
Corticosteroid	H02B0	Betamethasone and d-Chlorpheniramine Maleate	620531101
Corticosteroid	H02B0	Betamethasone and d-Chlorpheniramine Maleate	620531301
Corticosteroid	H02B0	Betamethasone and d-Chlorpheniramine Maleate	620531401
Corticosteroid	H02B0	Betamethasone and d-Chlorpheniramine Maleate	620531501
Corticosteroid	H02B0	Betamethasone and d-Chlorpheniramine Maleate	620531601
Corticosteroid	H02B0	Betamethasone acetate and Betamethasone sodium phosphate	642450087
Baricitinib	L04X0	Baricitinib	622573701
Baricitinib	L04X0	Baricitinib	622573801
Tocilizumab	L04C0	Ensitrelvir fumarate	620006184
Tocilizumab	L04C0	Tocilizumab (recombinant)	620006804
Tocilizumab	L04C0	Tocilizumab (recombinant)	620006805
Tocilizumab	M01C0	Tocilizumab (recombinant)	622242201
Tocilizumab	M01C0	Tocilizumab (recombinant)	622242301
Heparin	B01B1	Heparin calcium	620007487
Heparin	B01B1	Heparin calcium	620007488
Heparin	B01B1	Heparin calcium	620812201
Heparin	B01B1	Heparin calcium	620812203
Heparin	B01B1	Heparin calcium	621824702

Heparin	B01B1	Heparin calcium	621824802
Heparin	B01B1	Heparin calcium	621824902
Heparin	B01B1	Heparin calcium	621825002
Heparin	B01B1	Heparin calcium	621825102
Heparin	B01B1	Heparin calcium	621933401
Heparin	B01B1	Heparin calcium	622458001
Heparin	B01B1	Heparin calcium	643330003
Heparin	B01B1	Heparin calcium	620002286
Heparin	B01B1	Heparin calcium	620004183
Heparin	B01B1	Heparin calcium	620004184
Heparin	B01B1	Heparin calcium	620004185
Heparin	B01B1	Heparin calcium	620004186
Heparin	B01B1	Heparin calcium	620004187
Heparin	B01B1	Heparin calcium	620004188
Heparin	B01B1	Heparin calcium	620005699
Heparin	B01B1	Heparin calcium	620005700
Heparin	B01B1	Heparin calcium	620005701
Heparin	B01B1	Heparin calcium	620006312
Heparin	B01B1	Heparin calcium	620006725
Heparin	B01B1	Heparin calcium	620006726
Heparin	B01B1	Heparin calcium	620006727
Heparin	B01B1	Heparin calcium	620006728
Heparin	B01B1	Heparin calcium	620006729
Heparin	B01B1	Heparin calcium	620006734
Heparin	B01B1	Heparin calcium	620006739
Heparin	B01B1	Heparin calcium	620006740
Heparin	B01B1	Heparin calcium	620007489
Heparin	B01B1	Heparin calcium	620007490
Heparin	B01B1	Heparin calcium	620007491
Heparin	B01B1	Heparin calcium	620007492
Heparin	B01B1	Heparin calcium	620008392
Heparin	B01B1	Heparin calcium	620008393
Heparin	B01B1	Heparin calcium	620008395
Heparin	B01B1	Heparin calcium	620812504
Heparin	B01B1	Heparin calcium	621653101
Heparin	B01B1	Heparin calcium	621653201
Heparin	B01B1	Heparin calcium	621825301
Heparin	B01B1	Heparin calcium	621825302
Heparin	B01B1	Heparin calcium	621825401
Heparin	B01B1	Heparin calcium	621825501
Heparin	B01B1	Heparin calcium	621825502
Heparin	B01B1	Heparin calcium	621825602
Heparin	B01B1	Heparin calcium	621825701

Heparin	B01B1	Heparin calcium	621825704
Heparin	B01B1	Heparin calcium	621825802
Heparin	B01B1	Heparin calcium	621825901
Heparin	B01B1	Heparin calcium	621826004
Heparin	B01B1	Heparin calcium	621826102
Heparin	B01B1	Heparin calcium	621826301
Heparin	B01B1	Heparin calcium	621826402
Heparin	B01B1	Heparin calcium	621832801
Heparin	B01B1	Heparin calcium	621956701
Heparin	B01B1	Heparin calcium	622083701
Heparin	B01B1	Heparin calcium	622083801
Heparin	B01B1	Heparin calcium	622083901
Heparin	B01B1	Heparin calcium	622131301
Heparin	B01B1	Heparin calcium	622131401
Heparin	B01B1	Heparin calcium	643330011
Heparin	B01B2	Enoxaparin sodium	620006786
Heparin	B01B2	Dalteparin sodium	620006328
Heparin	B01B2	Dalteparin sodium	620007360
Heparin	B01B2	Dalteparin sodium	620812701
Heparin	B01B2	Dalteparin sodium	621671901
Heparin	B01B2	Dalteparin sodium	621673101
Heparin	B01B2	Dalteparin sodium	621673102
Heparin	B01B2	Dalteparin sodium	621673901
Heparin	B01B2	Dalteparin sodium	621699701
Heparin	B01B2	Dalteparin sodium	621699702
Heparin	B01B2	Dalteparin sodium	621702701
Heparin	B01B2	Dalteparin sodium	621702702
Heparin	B01B2	Dalteparin sodium	621757301
Heparin	B01B2	Dalteparin sodium	621757401
Heparin	B01B2	Dalteparin sodium	621994801
Heparin	B01B2	Dalteparin sodium	621994901
Heparin	B01B2	Dalteparin sodium	621995001
Heparin	B01B2	Dalteparin sodium	621995101
Heparin	B01B2	Dalteparin sodium	622757900
Heparin	B01B2	Parnaparin sodium	621480901
Heparin	B01B2	Parnaparin sodium	621480902
Heparin	B01B2	Parnaparin sodium	621515201
Heparin	B01B2	Parnaparin sodium	621728001
Heparin	B01B2	Parnaparin sodium	621728101
Heparin	B01B2	Parnaparin sodium	621728201
Heparin	B01B2	Parnaparin sodium	621989201
Heparin	B01B2	Parnaparin sodium	621989202
Heparin	B01B2	Parnaparin sodium	621989301

Heparin	B01B2	Parnaparin sodium	621989302
Heparin	B01B2	Parnaparin sodium	621989401
Heparin	B01B2	Parnaparin sodium	621989402
Heparin	B01B2	Parnaparin sodium	640408134
Heparin	B01B2	Reviparin sodium	620009151
Heparin	B01B2	Reviparin sodium	640463052
Heparin	B01B2	Reviparin sodium	640463139
Heparin	B01B3	Heparin sodium	620002282
Heparin	B01B3	Heparin sodium	620002283
Heparin	B01B3	Heparin sodium	620002284
Heparin	B01B3	Heparin sodium	620002285
Heparin	B01B3	Heparin sodium	620004192
Heparin	B01B3	Heparin sodium	620004193
Heparin	B01B3	Heparin sodium	620004194
Heparin	B01B3	Heparin sodium	620004195
Heparin	B01B3	Heparin sodium	620004761
Heparin	B01B3	Heparin sodium	620004762
Heparin	B01B3	Heparin sodium	620004763
Heparin	B01B3	Heparin sodium	620004764
Heparin	B01B3	Heparin sodium	620005186
Heparin	B01B3	Heparin sodium	621463101
Heparin	B01B3	Heparin sodium	621463103
Heparin	B01B3	Heparin sodium	621463201
Heparin	B01B3	Heparin sodium	621463203
Heparin	B01B3	Heparin sodium	621463401
Heparin	B01B3	Heparin sodium	621463403
Heparin	B01B3	Heparin sodium	621497401
Heparin	B01B3	Heparin sodium	621497403
Heparin	B01B3	Heparin sodium	621544001
Heparin	B01B3	Heparin sodium	621544005
Heparin	B01B3	Heparin sodium	621544101
Heparin	B01B3	Heparin sodium	621544105
Heparin	B01B3	Heparin sodium	621544201
Heparin	B01B3	Heparin sodium	621544205
Heparin	B01B3	Heparin sodium	621544301
Heparin	B01B3	Heparin sodium	621544305
Heparin	B01B3	Heparin sodium	621700002
Heparin	B01B3	Heparin sodium	621700102
Heparin	B01B3	Heparin sodium	621700202
Heparin	B01B3	Heparin sodium	622811101
Heparin	B01B3	Heparin sodium	640462020
Heparin	B01B3	Heparin sodium	640462021
Heparin	B01B3	Heparin sodium	640462023

Paxlovid	J05B6	Nirmatrelvir and Ritonavir	622967701
Paxlovid	J05B6	Nirmatrelvir and Ritonavir	622937801
Molnupiravir	J05B6	Molnupiravir	628785401
Ensitrelvir	J05B6	Ensitrelvir fumarate	628785801

Table S4. Medical conditions and associated codes identified by the Centers for Disease Control and Prevention (CDC) as risk factors for severe illness or death from COVID-19

Categories of medical conditions	Sub-category	ICD-10 Codes
Malignancies	Solid malignancy	C00-C80
	Hematological malignancy	C81-C96
Metabolic disorder	Type I DM	E10
	Type II DM	E11
	Obesity	out of scope
Cardiovascular	Cerebrovascular disease	I60-69
	Heart failure	I50
	Ischemic heart disease	I20-25
	Cardiomyopathy	I42
	Hypertension	I10, I11, I12, I13
Respiratory disease	Interstitial lung disease	J80-84
	Pulmonary embolism	I26
	Pulmonary hypertension	I27
	Bronchial asthma	J45
	Bronchiectasis	J47
	Chronic obstructive pulmonary disease	J44
	Tuberculosis infection	A15-19
	Cystic fibrosis	E84
	bronchopulmonary dysplasia	out of scope
Liver disease	Cirrhosis	K74
	Non-alcoholic fatty liver disease	K760
	Alcoholic liver disease	K70
	Autoimmune hepatitis	K754
	Hepatitis B	B180,181
	Hepatitis C	B182
Renal Disease	Chronic kidney disease with dialysis	N184, 185
	Chronic kidney disease without dialysis	N184, 185
Neuropsychiatric disorders	Mood disorder	F30-39
	Schizophrenia	F20-29
	Dementia	F00-02
Lack of exercise		out of scope
Smoking		out of scope
Pediatric		out of scope
Genetic disorder	Down syndrome	Q90
	Sickle cell disease	D57
	Alpha-1-antitrypsin deficiency	E88.01
	Thalassemia	D56
Immunocompromised condition	HIV infection	B20-24
	Organ transplantation	T86, Z94
	Primary immunodeficiency	D848
